# HIDTI: integration of heterogeneous information to predict drug-target interactions

**DOI:** 10.1038/s41598-022-07608-3

**Published:** 2022-03-08

**Authors:** Jihee Soh, Sejin Park, Hyunju Lee

**Affiliations:** School of Electrical Engineering and Computer Science, Gwangju Institute of Science and Technology, Gwangju, 61005 South Korea

**Keywords:** Data mining, Machine learning

## Abstract

Identification of drug-target interactions (DTIs) plays a crucial role in drug development. Traditional laboratory-based DTI discovery is generally costly and time-consuming. Therefore, computational approaches have been developed to predict interactions between drug candidates and disease-causing proteins. We designed a novel method, termed heterogeneous information integration for DTI prediction (HIDTI), based on the concept of predicting vectors for all of unknown/unavailable heterogeneous drug- and protein-related information. We applied a residual network in HIDTI to extract features of such heterogeneous information for predicting DTIs, and tested the model using drug-based ten-fold cross-validation to examine the prediction performance for unseen drugs. As a result, HIDTI outperformed existing models using heterogeneous information, and was demonstrating that our method predicted heterogeneous information on unseen data better than other models. In conclusion, our study suggests that HIDTI has the potential to advance the field of drug development by accurately predicting the targets of new drugs.

## Introduction

Drug development is a costly, time-consuming, and risky process with no guarantee of success^1^. Proteins are the main target class for drugs since drugs typically bind to target proteins to produce the desired therapeutic effect. As proteins linked to diseases are continuously being discovered, the identification of drugs targeting these disease-related proteins has become increasingly important. Thus, identifying drug-target interactions (DTIs; also known as compound-protein interactions) is now a critical step in the early stages of drug development and drug repositioning^2,3^. Recently, computational methods for accurately identifying potential DTIs have received significant attention^4^.

Existing methods for predicting DTIs include molecular docking- and machine learning-based models. Molecular docking-based methods have been used to predict DTIs by finding stable complexes with three-dimensional (3D) simulations^5–8^. Li et al.^6^ and Liu et al.^7^ provided comparative assessments of scoring functions for protein-ligand complexes to objectively evaluate the available scoring functions. Li et al.^8^ developed a web-based tool called TarFisDock to predict the possible binding proteins for a given ligand using docking methods. Using a docking-based inverse screening approach, Kumar et al.^9^ proposed the compound prioritization method by integrating machine learning, quantitative-structure activity relationship, and classical molecular docking approaches to identify probable hits. Their approach was based on the concept that molecules with better binding affinities should have the expected biological activity. In addition, Kinnings et al.^10^ developed a new server, ReverseScreen3D, that applies a reverse virtual screening method to find potential targets for a compound of interest. Such methods can be effective because they consider 3D structures. However, if the 3D structure is unknown, molecular docking-based methods cannot be applied.

Machine learning-based methods incorporate features of both the drug and protein to predict DTIs and learn the binding patterns of known drug-target pairs^4,11–13^. Yu et al.^11^ designed two powerful methods based on the random forest (RF) and support vector machine (SVM) algorithms using chemical, genomic, and pharmacological information from the DrugBank database. Faulon et al.^12^ proposed a model that predicts DTIs by using representations of proteins from their atomic structures.

Based on recent advances in deep learning, several DTI prediction methods have been developed using simple representations of drugs and proteins^14–16^. Tsubaki et al.^14^ proposed an end-to-end representation learning approach to predict interactions between drugs and targets, where a graph neural network was used to present drug structures and a convolutional neural network was used to represent protein sequences. Öztürk et al.^15^ reported a binding affinity prediction approach, called DeepDTA, based on convolutional neural networks using simple inputs for drugs and proteins. Gao et al.^16^ also used low-level representations for drugs and proteins to directly predict DTIs and provided biological insights from their predictions.

Network-based methods have also been developed for predicting DTIs^2,17–20^. These approaches incorporate complex relationships between heterogeneous drug and target information, such as drug-drug interactions (DDIs), protein-protein interactions (PPIs), drug or protein structure similarities, and relationships between drugs and side effects or diseases. Alaimo et al.^17^ used domain-dependent knowledge, including drug and target similarities, to predict DTIs. Kim et al.^18^ showed that DDIs and the side effects of drugs constitute useful information for predicting DTIs. Wang et al.^19^ proposed a heterogeneous network model, which involves collecting omics information about diseases, drugs, and drug targets to obtain closeness scores between diseases and drugs. However, this model was susceptible to deviations caused by noise and the high dimensionality of the heterogeneous data. To overcome this issue, Luo et al.^2^ developed a method called DTINet, which not only integrates heterogeneous information but also compensates for the complexity of large-scale high-dimensional biological data by learning informative low-dimensional feature vectors of drugs and proteins. NeoDTI was developed by Wan et al.^20^ as a further improvement in DTI prediction accuracy by learning topology-preserving representations from neighbor information in heterogeneous networks.

Although drug- and protein-related data can help to accurately predict DTIs, the previous approaches summarized above are only applicable to predictions for drugs with this information available. If there is insufficient information about a drug, as is often the case for newly developed drugs, these approaches are not helpful. To overcome this limitation, in this study, we aimed to develop an approach that can predict DTIs by learning feature vectors from heterogeneous information. Since additional information results in very large dimensions of feature vectors, it would not be suitable to use complex deep learning models, considering that the number of samples is insufficient. To solve these problems, we developed a new approach, termed heterogeneous information integration for DTI prediction (HIDTI), based on a residual network and classifier. First, we constructed deep neural network (DNN) models for feature generation, in which known heterogeneous information, including DDIs, PPIs, drug-side effect associations (DSIE), drug-disease associations (DDIS), and protein-disease associations (PDIS), were used to predict unknown heterogeneous information for unseen drugs. Second, we constructed a residual network-based model using skip connection to extract features from the heterogeneous information that was integrated to predict DTIs. The residual network is not complex, but was designed to extract features from high-dimensional vectors.

**Figure 1:**
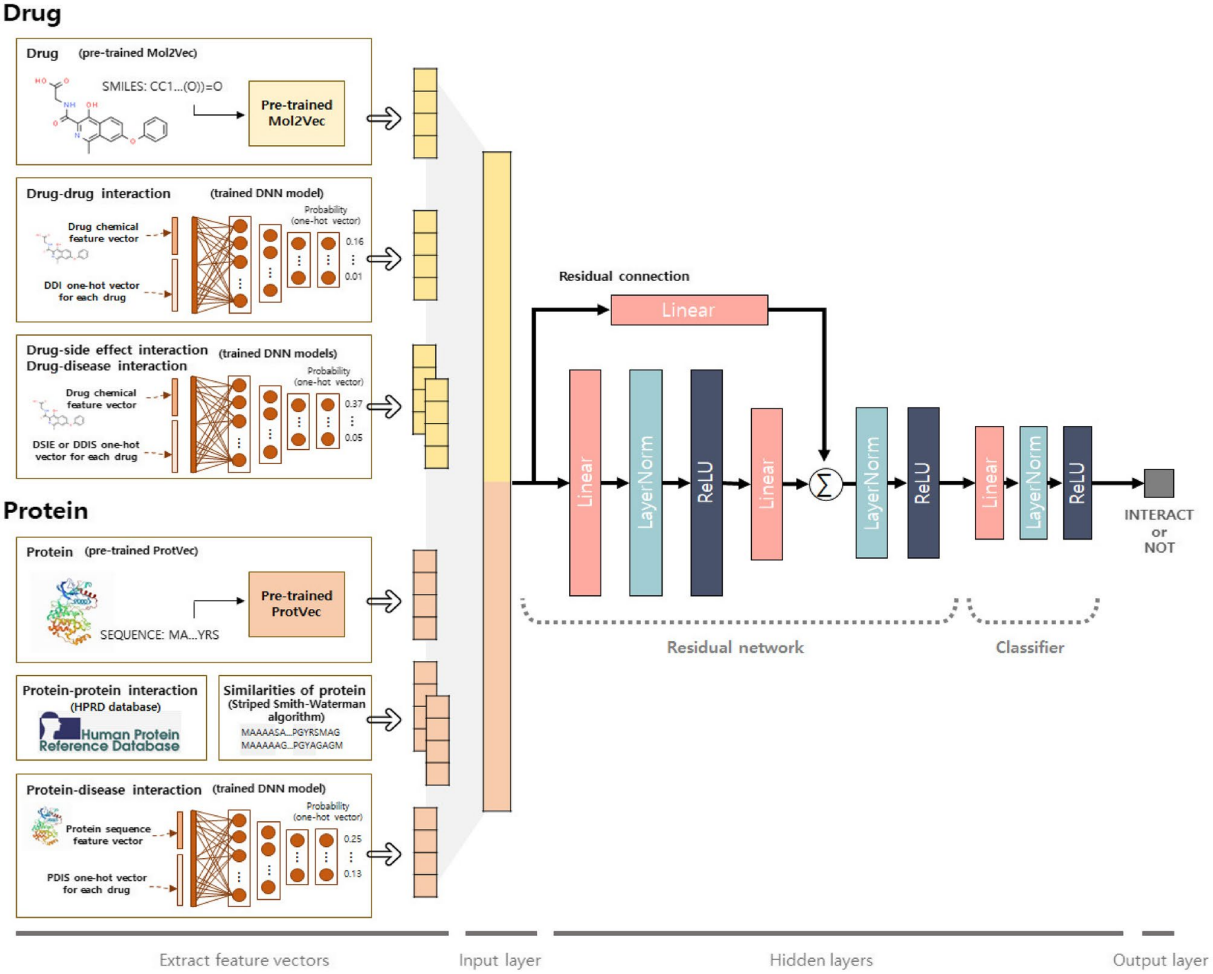
Architecture of the HIDTI model for predicting drug-target interactions (DTIs). Drug-related features include SMILES strings, drug-drug interactions (DDIs), drug-side effect associations (DSIE), and drug-disease associations (DDIS). Protein-related features include protein sequences, protein-protein similarities, protein–protein interactions (PPIs), and protein–disease interactions (PDIS). These are concatenated and fed into the neural network with a residual block. For unseen drugs, deep neural network (DNN) models are used to predict each item of heterogeneous information to obtain the input vectors of drug-target pairs. Ultimately, our model provides a binary output (1 or 0), considering the interaction between the drug and protein.

The performance of our model was tested using ten-fold cross-validation on drug-based folds for previously unseen drugs. The performance of previous approaches was also tested using cross-validations on DTI pair-based folds^2,19^, where the drugs can appear in both the training and test sets. Although these existing approaches have shown some utility in repositioning previously known drugs, they have not yet been tested for unseen drugs. An overview of the proposed model is presented in Fig. 1).

## Methods

### Datasets

We collected data on drugs and proteins from a previous study^20^ and removed duplicates. The details of the datasets are provided in Figs. S1–S4 in the Supplementary Materials. As a result, 707 drugs and 1489 proteins were used in our experiments. We represented the 707 drugs according to their DrugBank IDs in the form of simplified molecular-input line-entry system (SMILES) strings that included chemical structure information for molecules using short ASCII strings^21^. Specifically, the DrugBank ID of each drug was converted to the PubChem Compound ID (CID) and SMILES strings of drugs were extracted from the PubChem database^22^. We represented the 1489 proteins with UniProt IDs in the form of protein sequences that were extracted from the UniProtKB database (UniProt Consortium, 2019).

Because heterogeneous data related to proteins and drugs were included in the study by Wan et al.^20^, we also used these data to test the performance of our model for this specific context. Wan et al.^20^ extracted drug-protein interactions and DDIs from the DrugBank database (Version 3.0)^23^. PPIs were obtained from the Human Protein Reference Database (HPRD) (Release 9)^24^. Protein similarities were calculated using the pairwise Smith-Waterman scores^25^. Information associated with disease (drug-disease and protein-disease) and side effects (drug-side effects) was extracted from the Comparative Toxicogenomics Database (CTD)^26^ and SIDER database (Version 2)^27^, respectively.

A summary of the datasets used in our experiments is presented in Tables 1 and  2. For drugs, the minimum, maximum, and average lengths of SMILES strings were 3, 416, and 58, respectively. For proteins, the minimum, maximum, and average lengths were 38, 3608, and 371 amino acids, respectively. The details of drugs and proteins are described in Supplementary Materials and Figs. S1–S4.

**Table 1:** Dataset statistics.

# of drugs	# of proteins	# of side effects	# of diseases	Total
707	1489	4192	5603	11,991

**Table 2:** Positive interactions in our datasets.

Type of interaction	# of positives
Drug–protein	1909
Drug–drug	10,024
Drug-side effect	80,160
Drug-disease	199,022
Protein–protein	7133
Protein–disease	1,572,157

### Generating features of heterogeneous information

We constructed feature vectors for drug- and protein-related information. In previous studies, drug chemical and protein sequence feature vectors were constructed from SMILES strings and protein sequences, respectively^14–16^. Thus, we extracted drug chemical and protein sequence feature vectors from pre-trained Mol2vec^28^ and ProtVec^29^ models. These feature vectors can remove the length limitation of the drug and protein strings in the deep learning approach. We define drug chemical features as *Drug* for $n_d$ drugs and protein sequence features as *Protein* for $n_p$ proteins.

1 $$\begin{aligned}&Drug=\left[ {\begin{array}{*{20}l}\mathrm {Drug_1}^\intercal&\mathrm {Drug_2}^\intercal&\ldots&\mathrm {Drug_{n_{d}}}^\intercal \end{array} } \right]^\intercal , \end{aligned}$$

2 $$\begin{aligned}&Protein=\left[ {\begin{array}{*{20}l}\mathrm {Protein_1}^\intercal&\mathrm {Protein_2}^\intercal&\ldots&\mathrm {Protein_{n_{p}}}^\intercal \end{array} } \right]^\intercal , \end{aligned}$$

where $\mathrm {Drug_i}$ is a drug chemical feature vector of size 300 obtained using Mol2vec for the *i*-th drug, and $\mathrm {protein}_{i}$ is a protein sequence feature vector of size 100 obtained using ProtVec for the *i*-th protein.

Interactions between drugs and other drugs/side effects/disease are represented using one-hot vectors as follows:

3 $$\begin{aligned}&{DDI}=\left[ {\begin{array}{*{20}l}\mathrm {DDI_1}^\intercal&\mathrm {DDI_2}^\intercal&\ldots&\mathrm {DDI_{n_{d}}}^\intercal \end{array} } \right]^\intercal , \end{aligned}$$

4 $$\begin{aligned}&{DSIE}=\left[ {\begin{array}{*{20}l}\mathrm {DSIE_1}^\intercal&\mathrm {DSIE_2}^\intercal&\ldots&\mathrm {DSIE_{n_{d}}}^\intercal \end{array} } \right]^\intercal , \end{aligned}$$

5 $$\begin{aligned}&{DDIS}=\left[ {\begin{array}{*{20}l}\mathrm {DDIS_1}^\intercal&\mathrm {DDIS_2}^\intercal&\ldots&\mathrm {DDIS_{n_{d}}}^\intercal \end{array} } \right]^\intercal , \end{aligned}$$

where $\mathrm {DDI_i}$ is a DDI feature vector of size 707, $\mathrm {DSIE_i}$ is a DSIE feature vector of size 4192, and $\mathrm {DDIS_i}$ is a DDIS feature vector of size 5603 for each drug *i*. For training data, these features were obtained using the datasets as described in the preceding subsection. In addition, interactions between proteins and other proteins/diseases and protein similarities were represented using one-hot vectors as follows:

6 $$\begin{aligned} {PPI}=\left[ {\begin{array}{*{20}l}\mathrm {PPI_1}^\intercal&\mathrm {PPI_2}^\intercal&\ldots&\mathrm {PPI_{n_{p}}}^\intercal \end{array} } \right]^\intercal , \end{aligned}$$

7 $$\begin{aligned} {PSIM}=\left[ {\begin{array}{*{20}l}\mathrm {PSIM_1}^\intercal&\mathrm {PSIM_2}^\intercal&\ldots&\mathrm {PSIM_{n_{p}}}^\intercal \end{array} } \right]^\intercal , \end{aligned}$$

8 $$\begin{aligned} {PDIS}=\left[ {\begin{array}{*{20}l}\mathrm {PDIS_1}^\intercal&\mathrm {PDIS_2}^\intercal&\ldots&\mathrm {PDIS_{n_{p}}}^\intercal \end{array} } \right]^\intercal , \end{aligned}$$

where $\mathrm {PPI_i}$ is a PPI feature vector of size 1489, $\mathrm {PSIM_i}$ is a PSIM feature vector of size 1489, and $\mathrm {PDIS_i}$ is a PDIS feature vector of size 5603 for each protein *i*. We obtained the $\mathrm {PPI_i}$ and $\mathrm {PSIM_i}$ vectors from the HPRD and Smith-Waterman scores, respectively. In addition, for the $\mathrm {PDIS_i}$ vector, we obtained a feature vector from the CTD.

For unknown features in testing unseen drugs, we constructed DNN models to predict each feature vector for DDIs, DSIE, and DDIS, which are similar to the prediction model proposed by Wang et al.^30^. The details of our model are described in the Supplementary Materials. Wang et al.^30^ proposed a DNN model for predicting the adverse reactions of drugs using biological, biomedical, and drug chemical information. In this study, we modified this DNN model to predict various drug features as $F_{v}(x)$, $v\in \{DDI, DSIE, DDIS, PDIS\}$, in which the input x is the concatenated vector of the drug or protein and each item of heterogeneous information.

Each of the DNN models for predicting each vector of heterogeneous information, $F_{DDI}$, $F_{DSIE}$, and $F_{DDIS}$, consists of three fully connected layers with dimensions 1024, 512, and 128 for $F_{DDI}$, and dimensions 4096, 2048, and 1024 for $F_{DSIE}$ and $F_{DDIS}$. Input vectors are the drug chemical feature and each feature vector for DDIs, DSIE, and DDIS, and the outputs are each feature vector as follows:

9 $$\begin{aligned}&DDI=F_{DDI}([Drug; DDI]) \end{aligned}$$

10 $$\begin{aligned}&DSIE=F_{DSIE}([Drug; DSIE]) \end{aligned}$$

11 $$\begin{aligned}&DDIS=F_{DDIS}([Drug; DDIS]) \end{aligned}$$

Similarly, to predict PDIS, we also constructed a DNN model $F_{PDIS}$ consisting of three fully connected layers with dimensions 4096, 2048, and 1024 as follows:

12 $$\begin{aligned} PDIS=F_{PDIS}([Protein; PDIS]) \end{aligned}$$

To avoid overfitting in the training step, unique drugs and proteins were used for training, and we added a dropout layer with the dropout rate set to 0.5. The size of the last layer nodes for each model to predict DDI, DSIE, DDIS, and PDIS was 707, 4192, 5603, and 5603, respectively, which corresponded to the size of each feature vector in our dataset. In the training step, the values of the input feature vectors of DDI, DSIE, DDIS, and PDIS were based on one hot vector, and the models were trained to have the same output values as those of the input. In the testing step, the values of the input feature vectors of DDI, DSIE, DDIS, and PDIS were set to zero, and the predicted output feature vectors were used as feature vectors for unseen drugs in predicting DTIs.

### Residual network

Skip connections are helpful in improving the performance of DNNs by propagating a linear component^31^. ResNet^32^, using skip connection, was proposed to efficiently extract features of image data, and its huge success has led this architecture to become a basic and powerful concept. Transformer^33^ has achieved great performance in the field of natural language processing using DNNs with skip connections and attention without using recurrent or convolutional neural networks^34^. In the field of bioinformatics, Xia et al.^35^ used a skip connection approach by adding features in previous layers to the subsequent features for predicting growth rates of given cell lines and drug characteristics.

The skip connections in ResNet simply perform identity mapping, ${y=F(x)+x}$. Thus, there is no requirement for feature reduction^32^. However, a linear projection can be used to match the dimensions, ${y=F(x,\{W_i\})+W_{s}x}$, making it possible for shortcut connections to be used for feature selection^32^. There are several variants of the residual unit^33,36,37^. Srivastava et al.^36^ scaled *x* differently from *F*(*x*) in the residual block, ${y=F(x)+\lambda x}$, where ${\lambda }$ is usually greater than one. However, He et al.^37^ insisted that scaling causes difficulty for the gradient of the skip with respect to an exploding or vanishing gradient problem. Transformer^33^ uses layer normalization^38^, but with ${\lambda }$ set to 1, ${y=\mathrm {LayerNorm}(F(x)+x)}$. Liu et al.^31^ experimented with various residual unit forms and concluded that layer normalization could help to stabilize the optimization, and that setting ${\lambda }$ to 1 was preferable.

Considering this prior research, we built a residual network for feature selection, defined as follows:

13 $$\begin{aligned}&f(x, \{W_1, W_2\}) = W_2\mathrm {ReLu}(\mathrm {LayerNorm}(W_1x)) \end{aligned}$$

14 $$\begin{aligned}&y = \mathrm {ReLu}(\mathrm {LayerNorm}(f(x, \{W_1, W_2\}) + W_3x), \end{aligned}$$

where *x* is an ${M \times 1}$ feature vector, and ${W_1, W_2,}$ and ${W_3}$ are the ${M_1 \times M}$, ${M_2 \times M_1}$, and ${M_2 \times M}$ weight matrices, respectively. ReLu $=$max(0,x) is a rectified linear unit. In our experiment, the input vector had a large dimension. Thus, after each matrix multiplication for feature reduction, normalization was essential to stabilize the optimization.

### Prediction of DTIs

We constructed a residual network-based model for predicting DTIs using various feature vectors that contain heterogeneous information. The drug-related feature vector *D* and the protein-related feature vector *P* are defined as follows:

15 $$\begin{aligned} D=[Drug; DDI; DSIE; DDIS] \end{aligned}$$

16 $$\begin{aligned} P=[Protein; PPI; PSIM; PDIS] \end{aligned}$$

For a set of DTI pairs $I=\{(i_{drug}, i_{protein})\}$, we concatenated $D_{i_{drug}}$ and $P_{i_{protein}}$ (i.e., [$D_{i_{drug}}$; $P_{i_{protein}}$]), and fed them into the residual network. Then, a classifier with a single hidden layer predicts whether a drug and target interact. The hyperparameters of HIDTI included the number of residual blocks $\in$[1,2,3], number of hidden layers $\in$[1,2,3] for the classifier, and learning rate $\in$[$1\times 10^{-5}$, $1\times 10^{-4}$, $1\times 10^{-3}$, 0.01, 0.05, 0.1]. For hyperparameter optimization, we adopted a grid search algorithm, and the hyperparameters were determined using validation sets (for details, see Table S1 in the Supplementary Materials).

We used an early stopping strategy to avoid overfitting in the training step^39,40^. We used the ReLu function as the activation function. For the last layer, the sigmoid function S(x)$=$ $\frac{1}{1+e^{-x}}$ was used. Because DTI prediction is a binary prediction task (with both positive and negative interactions possible), binary cross entropy (BCE) was used as the loss function:

17 $$\begin{aligned} BCE=-t_{i}\log {(S(x))}-(1-t_i)\log {(1-S(x))}, \end{aligned}$$

where $t_i$ is the ground truth and S(x) is the predicted probability of a DTI.

The Adam algorithm was used to train the networks with the initial learning rate set to $1\times 10^{-5}$. In addition, a mini-batch size of 512 was used to update the weights of the network.

### Ten-fold cross-validation

In cross-validation, both training and test sets can contain the same drug if the sets are split based on drug-protein pairs. To evaluate the DTI prediction performance of the model for unseen drugs, we split the dataset into ten subsets based on the drugs. First, we counted the number of positive interactions between each drug and protein and sorted the drugs in descending order of counts. Second, we assigned the drugs to each fold so that the number of positive interactions was similar in each fold. To obtain negative samples in each fold, we randomly chose negative interactions between each drug and protein until the number of negative interactions was the same, three times, and five times that of the positive interactions. We randomly selected three negative interactions for certain drugs that did not exhibit any positive interactions because each drug interacted with an average of 2.7 proteins. Here, a negative interaction indicates any previously unreported interactions. In the ten-fold cross-validation, 85%, 5%, and 10% of the data were used for training, validation, and testing, respectively. The validation set was used for early stopping of the training.

### Method evaluation

We used the area under the receiver operating characteristic curve (AUC) between the true positive (TP) and false positive (FP) rates to evaluate the performance of our model. Three additional metrics were also used as performance measures: precision, recall, and F1-score. These metrics are calculated to evaluate predictive power according to four parameters, TP, true negative (TN), FP, and false negative (FN) rates, using the following equations:

18 $$\begin{aligned}&Precision = \frac{TP}{TP+FP} \end{aligned}$$

19 $$\begin{aligned}&Recall = \frac{TP}{TP+FN} \end{aligned}$$

20 $$\begin{aligned}&F1 score = \frac{2*Precision*Recall}{Precision+Recall} \end{aligned}$$

## Results

The performance of HIDTI was evaluated for both cases when heterogeneous information was available for the unseen drugs and when heterogeneous information could only be predicted for the unseen drugs. Because real-world DTIs are ordinarily imbalanced, we designed and conducted experiments for both balanced (positive:negative = 1:1) and imbalanced (positive:negative = 1:3 and 1:5) cases. The performance of the HIDTI model was then compared with that of other models according to the AUC value.

### Performance of HIDTI with available heterogeneous information for unseen drugs

We first consider the case in which existing (heterogeneous) drug- and protein-related information is available and used to predict unseen drugs. Table 3 shows the performance of the HIDTI model with available heterogeneous information for unseen drugs. First, as a baseline model, we predicted DTIs using only drug chemical and protein sequence feature vectors of drug-target pairs with a classifier consisting of two hidden layers. Using the balanced (positive:negative = 1:1) datasets, the average AUC value was 0.789, whereas the AUC values with unbalanced datasets (positive:negative = 1:3 and 1:5) were 0.879 and 0.853, respectively. Next, we conducted experiments for variants of HIDTI, where each drug- and protein-related feature was integrated with the features used in the baseline model. Each model predicted DTIs with a classifier consisting of two hidden layers, as in the baseline model. All variants of HIDTI were based on common drug chemical and protein sequence feature vectors. When each of the additional feature vectors of DDIs, DSIEs, DDIS, PPIs, PSIM, and PDIS were integrated, the prediction performance increased compared with that obtained when only drug chemical and protein sequence feature vectors were used. Among them, the PDIS features were the most informative, with the highest average AUC for the 1:3 dataset, followed by the 1:5 and 1:1 cases, respectively. When all of these features were integrated, HIDTI achieved the best and equal performance for the unbalanced (1:3 and 1:5) dataset cases, closely followed by the balanced (1:1) case.

**Figure 2:**
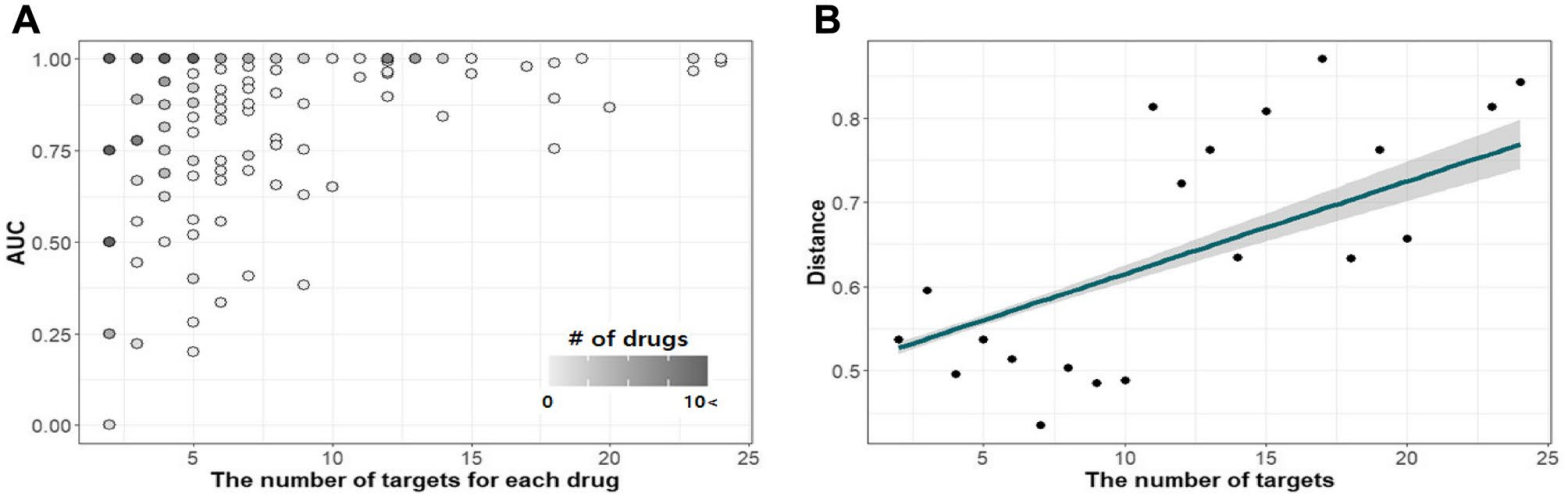
Performance evaluation of the HIDTI method for unseen drugs based on the number of targets. (**A**) The area under the receiver operating characteristic curve (AUC) values based on the number of targets for each drug. The shading intensity indicates the degree of the number of drugs with the corresponding AUC value. (**B**) Absolute values of the difference between the mean probabilities of positive and negative predicted interactions for each drug. This difference is denoted as the distance along the *y*-axis. Each dot represents the average distance of the drug for each number of targets.

Furthermore, we analyzed whether the number of targets for each drug was related to the performance of HIDTI when using a balanced dataset. In other words, for each unseen drug in the test datasets, we examined the AUC value according to the number of targets. We excluded drugs with a single target because the AUC values for these drugs could not be calculated. As a result, drugs with a large number of targets tended to have higher AUC values than those with fewer targets (Fig. 2A). In addition, the absolute value of the difference between the mean probabilities of positively and negatively predicted interactions, which is denoted as a distance in Fig. 2B, was measured for each drug. These distances were found to increase with an increase in the number of drug targets. Figure S5 shows that the mean probability values of positively predicted interactions become larger and those of negatively predicted interactions become smaller with an increasing number of targets. Also, the average standard deviation in probability values for positive and negative interactions of drugs has a decreasing trend with an increase in the number of targets (Fig. S5). Given the increase in distance values and trends in statistic values of probabilities, we could confirm that as the number of targets increases, the positive interactions tend to cluster well with other positives and negative interactions tend to cluster well with other negatives. Overall, these results showed that our HIDTI method could predict DTI pairs more accurately and stably when more targets interact with a given drug.

**Table 3:** Performance evaluation of HIDTI and other models for when heterogeneous information was available for unseen drugs.

Methods	Ratio of positive and negative interactions											
	1:1				1:3				1:5			
	AUC	Precision	Recall	F1	AUC	Precision	Recall	F1	AUC	Precision	Recall	F1
Baseline	0.789	0.693	0.816	0.741	0.879	0.732	0.708	0.716	0.853	0.670	0.607	0.630
Single info												
+DDI	0.832	0.720	0.804	0.758	0.890	0.734	0.741	0.734	0.873	0.669	0.637	0.649
+DSIE	0.816	0.719	0.792	0.752	0.881	0.675	0.772	0.714	0.867	0.639	0.616	0.621
+DDIS	0.834	0.723	0.817	0.766	0.876	0.668	0.760	0.707	0.866	0.602	0.635	0.615
+PPI	0.832	0.721	0.837	0.767	0.893	0.745	0.752	0.747	0.891	0.732	0.659	0.690
+PSIM	0.872	0.750	0.848	0.795	0.911	0.802	0.758	0.778	0.901	0.730	0.694	0.709
+PDIS	0.892	0.811	0.821	0.815	0.921	0.811	0.767	0.786	0.913	0.749	0.720	0.733
Multiple info												
HIDTI (Ours)	**0.919**	**0.852**	**0.849**	**0.850**	**0.936**	**0.834**	**0.793**	**0.810**	**0.936**	**0.800**	**0.759**	**0.778**

The baseline model represents the prediction of DTIs using only drug chemical and protein sequence feature vectors.

The best performance values are in [bold].

### Performance of HIDTI with predicted heterogeneous information for unseen drugs

We next assessed the prediction of DTIs when drug- and protein-related information was not available, and thus had to be predicted for unseen drugs. In cases where the relationship between diseases and proteins was also unknown, we extracted protein-disease feature vectors using the predictive model. To generate predicted feature vectors, we trained DNN models for DDIs, DSIEs, DDIS, and PDIS with each dataset as described in the Datasets subsection of the Methods.

Table 4 shows that even when predicted vectors were used, the prediction performances were similar to those obtained using existing known features. Consistently, when each feature was integrated with drug chemical and protein sequence feature vectors, the prediction performance increased. When all features were integrated, HIDTI achieved the highest average AUCs for the unbalanced datasets (1:5 and 1:3), closely followed by the balanced dataset (1:1 positives:negatives) with available PDIS features, and a similar pattern was found with all predicted features, although the AUCs were slightly lower for all dataset cases. The performance under this scenario was 0.021, 0.035, and 0.032 lower for the 1:1, 1:3, and 1:5 case, respectively, with available PDIS features, and was 0.030, 0.042, and 0.044 lower, respectively, with all predicted features, compared with that obtained when all existing features were used. These results demonstrated that the prediction performances of DTIs based on integrating each feature tended to decrease slightly when using predicted features rather than existing features in the case of balanced positive and negative interactions. In unbalanced cases, although the results were generally similar, predicted PDIS features seemed to have a critical impact on the decrease of DTI prediction performance (from 0.921 to 0.889 for the 1:3 case and from 0.913 to 0.881 for the 1:5 case) compared with the use of existing PDIS features. Thus, the decreased performance of HIDTI with predicted features might be mostly driven by predicted protein-disease relationships.

We also compared HIDTI with NeoDTI^20^, a graph-based method that uses heterogeneous information for DTI predictions, as NeoDTI has been proven to outperform several other methods. We also used the drug-based folds described in the “Ten-fold cross-validation” section to run this experiment under the same conditions as those used for evaluation of the performance of HIDTI itself. In this performance assessment of NeoDTI (see the Supplementary Materials for further details), the interacting edges of the test drugs, which represent heterogeneous drug-related information in the network, were set to zeroes in the training process, and the predicted values between proteins and test drugs were used for performance evaluation. For the HIDTI model, the predicted drug-related heterogeneous information was used for testing. As shown in Table 4, the average AUC value for NeoDTI was the highest for the 1:5 unbalanced dataset, followed by the balanced (1:1) dataset, and the lowest value was obtained for the unbalanced 1:3 case. HIDTI significantly outperformed NeoDTI for DTI prediction using predicted drug-related vectors for unseen drugs, with a *p*-value of $1.13\times 10^{-4}$, $2.55\times 10^{-4}$, and $2.69\times 10^{-3}$ for the 1:1, 1:3, and 1:5 cases, respectively, based on the *t*-test of AUC values of the ten folds.

To investigate the reason for this superior performance of HIDTI compared with that of NeoDTI, we further compared the prediction performance of the two models with drug-related heterogeneous information. HIDTI showed significantly better prediction ability than NeoDTI for all features, except for DDIs (Table 5). The prediction performance of each model for the cases using imbalanced datasets (1:3 and 1:5) was similar to that obtained using balanced datasets (Table 5). This result clarified that the superior performance of HIDTI in predicting drug-related heterogeneous information contributed to its better DTI prediction performance compared with that of NeoDTI.

**Table 4:** Performance evaluation of HIDTI and other models when heterogeneous information was predicted for unseen drugs.

Methods	Ratio of positive and negative interactions											
	1:1				1:3				1:5			
	AUC	Precision	Recall	F1	AUC	Precision	Recall	F1	AUC	Precision	Recall	F1
Baseline	0.789	0.693	0.816	0.741	0.879	0.732	0.708	0.716	0.853	0.670	0.607	0.630
Single info												
+DDI	0.812	0.695	0.814	0.746	0.878	0.716	0.727	0.718	0.868	0.701	0.617	0.655
+DSIE	0.824	0.740	0.780	0.757	0.866	0.728	0.703	0.712	0.854	0.650	0.627	0.635
+DDIS	0.808	0.704	0.784	0.740	0.853	0.664	0.725	0.691	0.844	0.641	0.609	0.623
+PPI	0.849	0.766	0.801	0.780	0.896	0.750	0.751	0.747	0.890	0.733	0.654	0.689
+PSIM	0.863	0.798	0.791	0.793	**0.911**	0.798	0.761	0.775	0.901	0.736	0.675	0.701
+PDIS	0.888	0.733	0.802	0.762	0.889	0.744	0.739	0.738	0.881	0.667	0.659	0.657
Multiple info												
HIDTI (predicted all)	0.889	0.831	0.818	0.823	0.894	0.797	0.771	0.781	0.892	0.765	0.697	0.727
HIDTI (available PDIS)	**0.898**	**0.856**	**0.820**	**0.837**	0.901	**0.799**	**0.781**	**0.787**	**0.904**	**0.770**	**0.717**	**0.740**
NeoDTI	0.828	0.566	0.651	0.758	0.809	0.622	0.646	0.629	0.841	0.497	0.583	0.605

The case of ‘HIDTI (available PDIS)’ refers to the use of existing protein-disease relationship (PDIS) features from our dataset, and the case of ‘HIDTI (predicted all)’ refers to the use of all predicted features from each deep neural network model described in “Generating features of heterogeneous information” section.

The best performance values are in [bold].

**Table 5:** Prediction performance with drug-related heterogeneous information for NeoDTI and HIDTI.

Drug related information	Ratio of positive and negative interactions					
	1:1		1:3		1:5	
	AUC					
	NeoDTI	HIDTI	NeoDTI	HIDTI	NeoDTI	HIDTI
DDI	**0.979**	0.678	**0.982**	0.678	**0.980**	0.679
DSIE	0.494	**0.845**	0.495	**0.845**	0.538	**0.845**
DDIS	0.525	**0.830**	0.508	**0.830**	0.499	**0.831**

The best performance values are in [bold].

### Comparison of HIDTI and machine learning algorithms

We also compared our model with other machine learning models, including SVM and RF classifiers (Tables S2–S5). For SVM, heterogeneous information helped to improve the prediction of DTIs in the case of available heterogeneous information for the unseen drugs although the prediction of DTIs was not significantly affected by predicted heterogeneous information. As a result, HIDTI outperformed the SVM models in all cases. For the RF models, the performance improvement by heterogeneous information was very small even though the prediction performance of the RF model was slightly higher than that of the HIDTI model. Thus, it is necessary to investiagte whether these results are due to the high performance of the baseline RF model or whether the RF model did not efficiently incorporate the relevant information when heterogeneous information was added.

### Performance evaluation after removing redundant DTIs

To examine the effect of redundant DTIs that could potentially inflate prediction performance, we evaluated the prediction performance again after removing similar drugs in test datasets so that the chemical structural similarities between drugs in the training and test datasets were all less than 0.6 (Table S6). After removing redundant DTIs, heterogeneous information was helpful for DTI predictions compared with the baseline, and the performance of HIDTI was still superior to that of NeoDTI. Moreover, after removing redundant DTIs, HIDTI still outperformed SVM for all cases and showed better AUC values compared to the RF model when heterogeneous information was available (Table S7).

### Ablation models of HIDTI to examine the impact of heterogeneous information

Given the apparent influence of heterogeneous information on model performance, we performed ablation studies to identify the specific influence of heterogeneous features on HIDTI in which input features were used after excluding heterogeneous information from the HIDTI. We also evaluated the performance of the models in which all drug-related or protein-related information was removed. The results showed that all protein-related information had the highest impact on HIDTI performance, and DDIs had the lowest impact on performance (Tables S9 and S10). This finding suggests that protein-related information is more useful in predicting DTIs in drug-based folds than drug-related information.

### Performance of models using randomly divided DTI pairs

We additionally performed experiments using randomly divided folds for ten-fold cross-validation based on DTI pairs, which is the same cross-validation approach used in the NeoDTI method. All positive DTI pairs and the same number of randomly selected negative DTI pairs were used to divide each fold. For the test sets, existing heterogeneous features were used for both NeoDTI and HIDTI, as this was the condition used in the original NeoDTI study^20^. This case represents a situation in which a drug in the training set can be included in the test set.

Both methods showed high performance in ten-fold cross-validation. The average AUCs for HIDTI were 0.99916, 0.99804, and 0.99771 for the 1:1, 1:3, and 1:5 datasets, respectively. Similarly, the AUCs of NeoDTI were 0.99971, 0.99430, and 0.99588 for the 1:1, 1:3, and 1:5 datasets, respectively. This similarity appears to be related to the fact that many drugs overlap each fold. This implies that both models can accurately predict DTIs when some drug targets are previously known in the training model.

### Model predictability

Many false positives were found to be involved in the proteins interacting with several drugs among DTI pairs in the test datasets. Thus, we suspected that the high performance of HIDTI might be attributed to the fact that proteins interacting with many drugs in the training set were predicted to interact with drugs in the test dataset. To test this possibility, we measured the baseline performance when a protein is predicted to interact with any drug in the test dataset if the ratio of positive interactions of a given protein in the training dataset is greater than or equal to 25%, 50%, or 75%, respectively. We refer to these three baseline cases as THR_25%, THR_50%, and THR_75%. For this evaluation, we compared the performance of HIDTI with that of NeoDTI based on the area under the precision recall curve (AUCPR), precision, recall, and F1-score. These measures were selected because they are considered to be useful metrics for imbalanced datasets.

**Figure 3:**
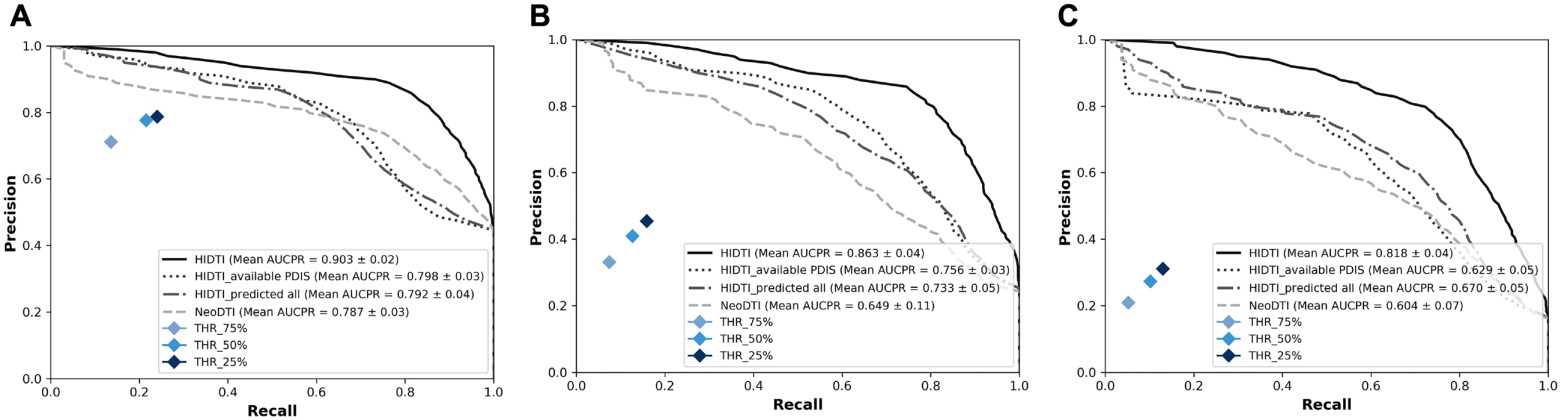
Performance evaluation of HIDTI and NeoDTI in terms of the AUCPR scores for balanced (1:1) (**A**) and imbalanced (1:3 and 1:5) (**B, C**) datasets. The three points represent the average precision and recall values for THR_25%, THR_50%, and THR_75% on drug-based ten-fold cross-validation.

Figure 3 shows the performance evaluation of each case where the ratio of positive and negative interactions is balanced (1:1) and imbalanced (1:3 and 1:5). For the balanced dataset, the average (± standard deviation) AUCPR scores for HIDTI, HIDTI_available PDIS, HIDTI_predicted all, and those for NeoDTI were 0.903 (± 0.02), 0.798 (± 0.03), 0.792 (± 0.04), and 0.787 (± 0.03), respectively. Although the overall performance decreased gradually with more predicted information, similar patterns were obtained for imbalanced datasets, with AUPRC scores of 0.863 (± 0.04), 0.756 (± 0.03), 0.733 (± 0.05), and 0.649 (± 0.11) in the case of the 1:3 dataset, and 0.818 (± 0.04), 0.629 (± 0.05), 0.670 (± 0.05), and 0.604 (± 0.07) in the case of the 1:5 dataset, respectively. The average precision and recall values for THR_25%, THR_50%, and THR_75% are also shown in Fig. 3. In the case of the balanced dataset, the best F1-scores of 86.93%, 77.23%, and 77.75% were achieved by HIDTI, HIDTI_available PDIS, and HIDTI_predicted all, respectively, and the best F1-score obtained for NeoDTI was 79.22%. However, the scenarios of THR_25%, THR_50%, and THR_75% resulted in significantly inferior performance with F1-scores of 42.11%, 40.08%, and 30.45%, respectively. For imbalanced cases, HIDTI, HIDTI_available PDIS, and HIDTI_predicted all achieved 86.55%, 77.65%, 76.26% F1-scores for the 1:3 dataset, and 80.58%, 71.15%, 73.10% for the 1:5 dataset, respectively. NeoDTI obtained an F1-score of 76.98% for the 1:3 dataset and an F1-score of 72.22% for the 1:5 dataset. Similar to the results above, under the scenarios of THR_25%, THR_50%, and THR_75% the F1-scores were markedly reduced to 38.17%, 30.83%, 22.41% for the 1:3 dataset, and to 24.83%, 19.53%, and 11.24% for the 1:5 dataset, respectively. Thus, the performances of all models decreased in imbalanced cases. However, through these results, we confirmed that our three models generally predicted unknown DTI pairs better than the existing model.

Finally, we investigated whether false positive interactions with high probabilities predicted by our model were potential true interactions. As an example, we focused on the dopamine receptor proteins D1A, D2, D3, D4, and D1B with 23, 36, 17, 14, and 12 interacting drugs, respectively, in the dataset. Table 6 shows the top 10 dopamine receptor protein-related DTI pairs with high prediction probabilities in the test datasets, along with the original labels in the dataset. Among these, only one was a negative interaction (orphenadrine). Cheng et al.^41^ analyzed the interaction mechanisms of various drugs (including cocaine, dopamine, amphetamine, and orphenadrine) with human dopamine transporters through computational and experimental methods. Since we could not find any report on the direct interaction between orphenadrine, which is used for the treatment of musculoskeletal pain and discomfort, and dopamine d1 receptor, further investigation might be needed to clarify their potential indirect relationship. Additionally, we divided DTI pairs based on protein classes, including Enzyme, Transporter, G-protein coupled receptor, Voltage-gated ion channel, and Transcription factor, from The Human Protein Atlas^42^ for unseen drugs. Table S11 shows the top 10 probabilities of false positive DTIs for unseen drugs according to the protein classes. Since there is a possibility that there may be new DTIs, we searched the literature for these 10 pairs of false positives. There is no reported evidence on direct interactions of these pairs; however, we expect that these false positive DTI pairs with high probabilities could be potential candidates for DTIs.

**Table 6:** Prediction probability of DTI pairs related to dopamine receptors.

Drug name	Gene name	Probability	Label
Ropinirole	DRD1	0.996647	1
Ziprasidone	DRD2	0.996076	1
Olanzapine	DRD3	0.995723	1
Thiothixene	DRD1	0.995303	1
Ropinirole	DRD3	0.995222	1
Ziprasidone	DRD3	0.993949	1
Orphenadrine	DRD1	0.993747	0
Risperidone	DRD3	0.993418	1
Perphenazine	DRD2	0.99307	1
Chlorpromazine	DRD2	0.992936	1

The probability represents the prediction values of DTI pairs in the test datasets, and the label represents the original labels of the DTI pairs in the dataset used in this study.

## Discussion and conclusion

Predicting DTIs is an essential task in drug discovery and development, and can further help in elucidating the mechanisms of biological processes related to drugs. In this study, we developed the HIDTI model to predict DTIs using diverse information related to drugs and proteins. In contrast to the majority of previous DTI prediction studies that measured the performance of their models by randomly selecting interacting pairs between drugs and targets, we measured model performance for interactions between unseen drugs and targets. The trained HIDTI model could accurately predict DTI pairs for unseen drugs based only on drug SMILES strings and protein sequences, as our model enables predicting drug- and protein-related heterogeneous features. Thus, the accuracy of the predicted feature vectors is important. Accordingly, the performance of HIDTI will be further improved if the predicted feature vectors contain more accurate information on drugs or proteins.

Although we did not perform an experiment for unseen proteins, the proposed model can also predict DTIs for such a case. These findings will also be useful for repurposing drugs for lesser-known proteins.

Our study focused on the use of binary classification to predict DTIs because we obtained DTI and heterogeneous data from network-based research, where edges between drugs and target interactions are represented in binary form. Other heterogeneous information related to drugs or proteins is also typically represented in binary form. Accordingly, many methods for DTI prediction have been developed for binary classification^2,3,11,12,14,16,20^. However, in the actual datasets, the number of positive interactions between drugs and targets was much smaller than the number of negative interactions, because the negatives included non-interacting pairs and unseen pairs of drugs and targets. Such imbalance in the data causes several problems such as overfitting. Thus, many approaches have been proposed to handle these problems, such as undersampling or oversampling. As an alternative to binary classification, DTIs can be predicted according to the binding strength between a drug and its targets using datasets such as Davis, Metz, and Kinase Inhibitor Bioactivty datasets^43–45^. However, it is difficult to determine a threshold for strong interactions, and the thresholds might depend on the specific drugs and datasets considered. We plan to apply the HIDTI model to binding strength-based DTI datasets in future work to address these questions.

In conclusion, our study suggests that HIDTI has the potential to advance the field of drug development by predicting the targets of new drugs.

## Supplementary Information

Supplementary Information.

## Data Availability

The datasets analyzed in the current study are available in the HIDTI repository: http://github.com/DMCB-GIST/HIDTI.
