# Supplementary material for: HIDTI: integration of heterogeneous information to predict drug-target interactions
Source: Sci Rep. 2022 Mar 8;12:3793. doi: 10.1038/s41598-022-07608-3 (PMC8904809; doi:10.1038/s41598-022-07608-3)
Supplement: Supplementary file 1 — Supplementary Information. [file 41598_2022_7608_MOESM1_ESM.pdf]

Supplementary Materials for

HIDTI: Integration of heterogeneous information to predict drug-target interactions

Jihee Soh, Sejin Park, and Hyunju Lee*

Gwangju Institute of Science and Technology, School of Electrical Engineering and Computer Science, Gwangju, Korea
*hyunjulee@gist.ac.kr

1. Materials and Methods

1.1 Data description

The datasets used in our experiments are summarized in Figures S1–S4. The distributions of the SMILES string and protein sequence lengths for drugs and proteins are shown in Figures S1 and S2. For the drugs, the minimum, maximum, median, and average lengths of the SMILES strings were 3, 416, 49, and 57.55, respectively. For proteins, the minimum, maximum, median, and average lengths were 38, 3608, 291, and 371, respectively.

Figure S3 shows the drug class information. Drugs were divided into 14 classes according to the first level of the Anatomical Therapeutic Chemical (ATC) classification system by the World Health Organization (WHO). The related information was extracted from the DrugBank database[1]. Figure S4 shows the five protein categories of our data: Enzyme, Transporter, G-protein coupled receptor, Voltage-gated ion channel, and Transcription factor. These classifications were obtained from The Human Protein Atlas[2].

The positive drug-target interaction pairs were obtained from the DrugBank database[1], which contains information on the pharmacological action and interaction types that are annotated as an antagonist, agonist, substrate, inhibitor, and inducer. The proteins in the positive interaction pairs can be further classified into drug targets, enzymes, carriers, and transporters.

1.2 Models for unseen drugs

1.2.1 Deep neural network (DNN) model for predicting feature vectors of heterogeneous information

To obtain heterogeneous information from unseen drugs, we adopted the DNN model proposed by Wang et al.[3]. This DNN model was designed to predict adverse drug reactions using biological, biomedical, and drug chemical information. The features for training and testing were slightly different. In the training step, they used biological, biomedical, and drug chemical information with adverse drug reaction features of the drugs, whereas these adverse drug reaction features were set to zero in the test step. The dimensions of each feature were 400, 1048, 1048, 15, and 1325 for drug, biological, biomedical, drug chemical, and adverse drug reactions, respectively. The hidden layers consisted of [1024, 512, 128] nodes and the output layer included 1325 nodes, which matched the size of the adverse drug reaction vector. Based on the concept of the DNN model proposed by Wang et al.[3], we designed our DNN models for each item of heterogeneous information, including drug-drug interaction (DDI), drug-side

---

[1] Knox, Craig, et al. "DrugBank 3.0: a comprehensive resource for 'omics' research on drugs." *Nucleic acids research* 39.suppl_1 (2010): D1035-D1041.

[2] Pontén, Fredrik, Karin Jirström, and Matthias Uhlen. "The Human Protein Atlas—a tool for pathology." *The Journal of Pathology: A Journal of the Pathological Society of Great Britain and Ireland* 216.4 (2008): 387-393.

[3] Wang, Chi-Shiang, et al. "Detecting potential adverse drug reactions using a deep neural network model." *Journal of medical Internet research* 21.2 (2019): e11016.

effect association (DSIE), and drug-disease association (DDIS). The drug vector with a size of 300 and each item of heterogeneous information, including DDI (707), DSIE (4192), and DDIS (5603), were concatenated, and the concatenated vectors were then used as the input vectors. The hidden layers consisted of [1024, 512, 128] nodes for the DDI vector prediction model, and [4096, 2048, 1024] for the DSIE and DDIS vector prediction models. The size of the output layers for each model was equal to the size of each heterogeneous vector. The objective function of each model is described as follows:

$$\text{Loss} = -\frac{1}{\text{N}}\sum_{i=1}^{N} y_i \log \hat{y} + (1 - y_i)\log(1 - \hat{y}),$$

where N is the output vector size, y_i is the actual binary value, and $\hat{y}$ is the predicted value. To avoid overfitting, we used unique drugs for training and added a dropout layer that was set to 0.5.

1.2.2 NeoDTI

To evaluate the performance of NeoDTI for unseen drugs, we modified the code of NeoDTI on Github (https://github.com/FangpingWan/NeoDTI). Because our folds consisted of drug-based folds, we set the interacting edges of the network to zero for the test drugs in the training process. The interacting edges represent heterogeneous drug-related information, including the DDI, DSIE, and DDIS, in the network. We then used the predicted values between proteins and test drugs for performance evaluation. The modified NeoDTI code is available at Github (https://github.com/DMCB-GIST/HIDTI).

1.3 Hyperparameters for optimizing HIDTI

For hyperparameter tuning, we used a grid-search algorithm. The number of residual blocks was assessed for $\in$[1,2,3], the number of hidden layers for the classifier was assessed for $\in$ [1,2,3], and the learning rate was assessed for $\in$[1×10^{-5}, 1×10^{-4}, 1×10^{-3}, 0.01, 0.05, 0.1].

The results of the grid-search algorithm are presented in Table S1. Finally, one residual block, one hidden layer for the classifier, and a learning rate of 1×10^{-5} were set for our model.

2. Performance of HIDTI based on of drug categories for unseen drugs

We additionally examined the performance of HIDTI based on drug categories for unseen drugs (Table S8). The prediction performance of drugs was the highest for systemic hormonal preparations; however, this was because the number of interactions to calculate the area under the receiver operating characteristic curve (AUC) values was only 10, and the highest AUC of one interaction affected the average AUC. Thus, the prediction performance of drugs targeting the nervous system had very high predictability, whereas drugs with anti-infective properties for systemic use had the lowest predictability.

Supplementary Figures and Tables

Figures

Figure S1. Distribution of SMILES string lengths

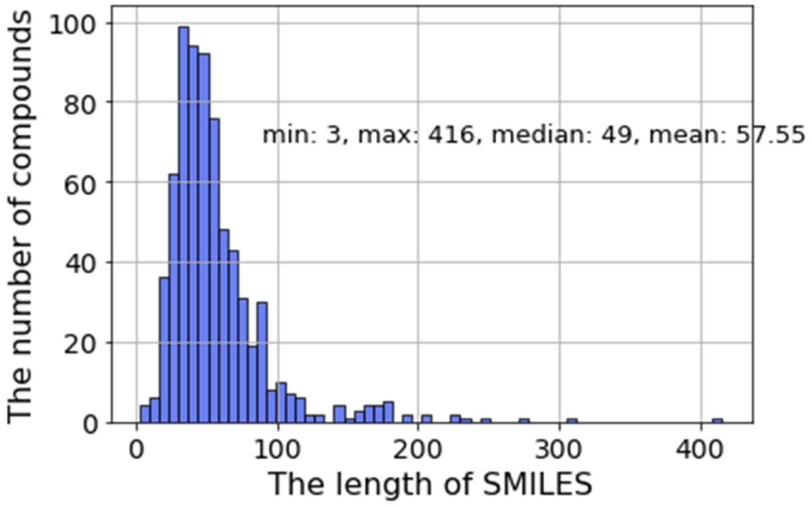

Figure S2. Distribution of protein sequence lengths

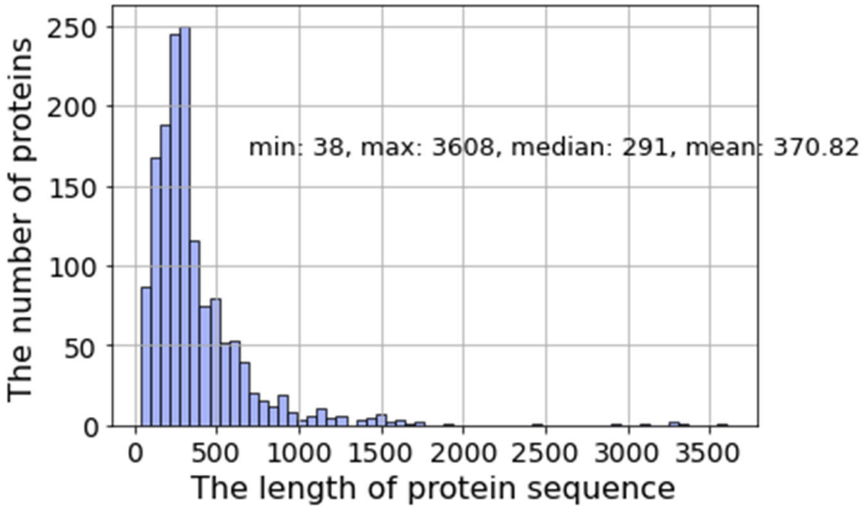

Figure S3. Summary of drugs in this study. The information includes the number of drugs in the ATC classification.

Code	Contents	# of drugs
A	Alimentary tract and metabolism	51
B	Blood and blood forming organs	12
C	Cardiovascular system	83
D	Dermatologicals	12
G	Genito-urinary system and sex hormones	21
H	Systemic hormonal preparations, excluding sex hormones and insulins	5
J	Antiinfectives for systemic use	74
L	Antineoplastic and immunomodulating agents	64
M	Musculo-skeletal system	27
N	Nervous system	151
P	Antiparasitic products, insecticides and repellents	14
R	Respiratory system	23
S	Sensory organs	15
V	Various	12

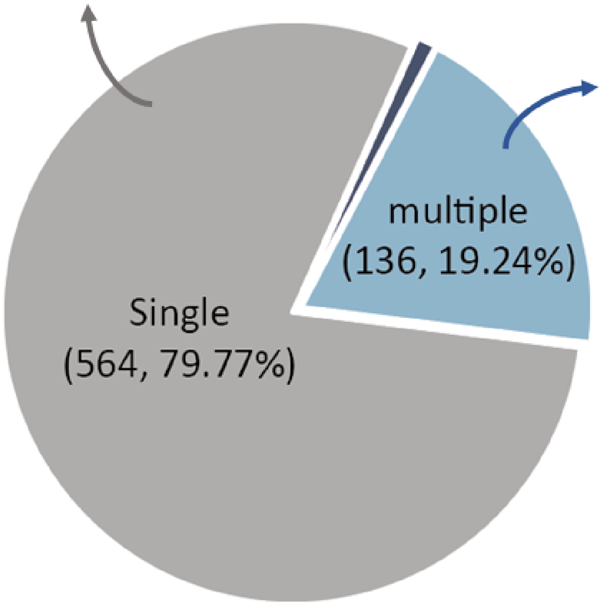

# of classes	# of drugs
2	92
3	27
4	7
5	7
6	2
7	1
8 - 14	0

Figure S4. Summary of proteins in this study. The information includes the number of proteins in five classes.

# of classes	# of proteins
2	91
3	1

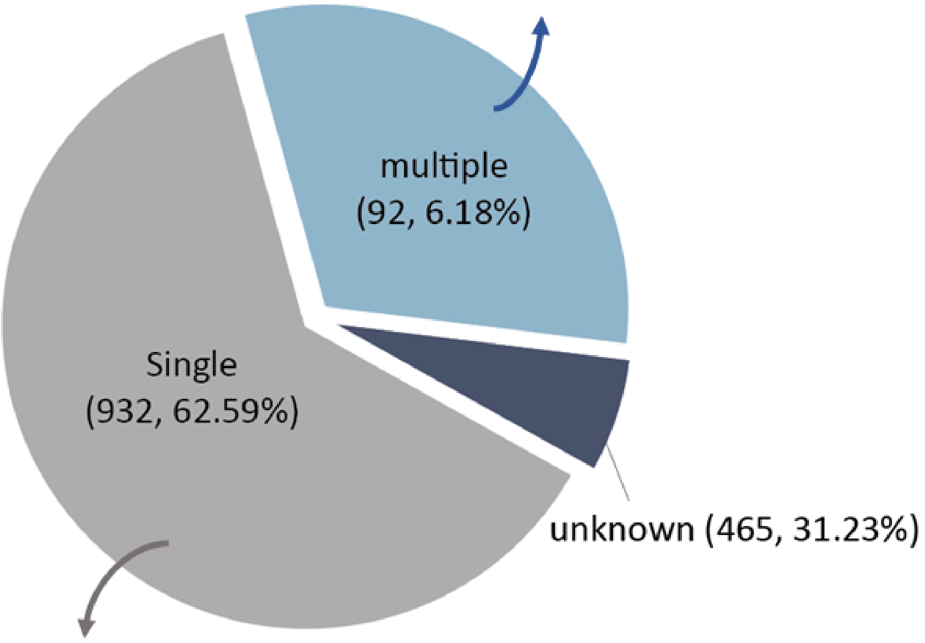

Class	# of proteins
Enzyme	688
Transporter	124
G-protein coupled receptor	68
Voltage-gated ion channel	10
Transcription factor	42

Figure S5. The average mean and standard deviation of probabilities for positive and negative interactions of drugs according to the number of targets.

A. The mean probabilities of negative interactions. B. The mean probabilities of positive interactions. C. The standard deviation of probabilities for negative interactions. D. The standard deviation of probabilities for positive interactions according to the number of targets.

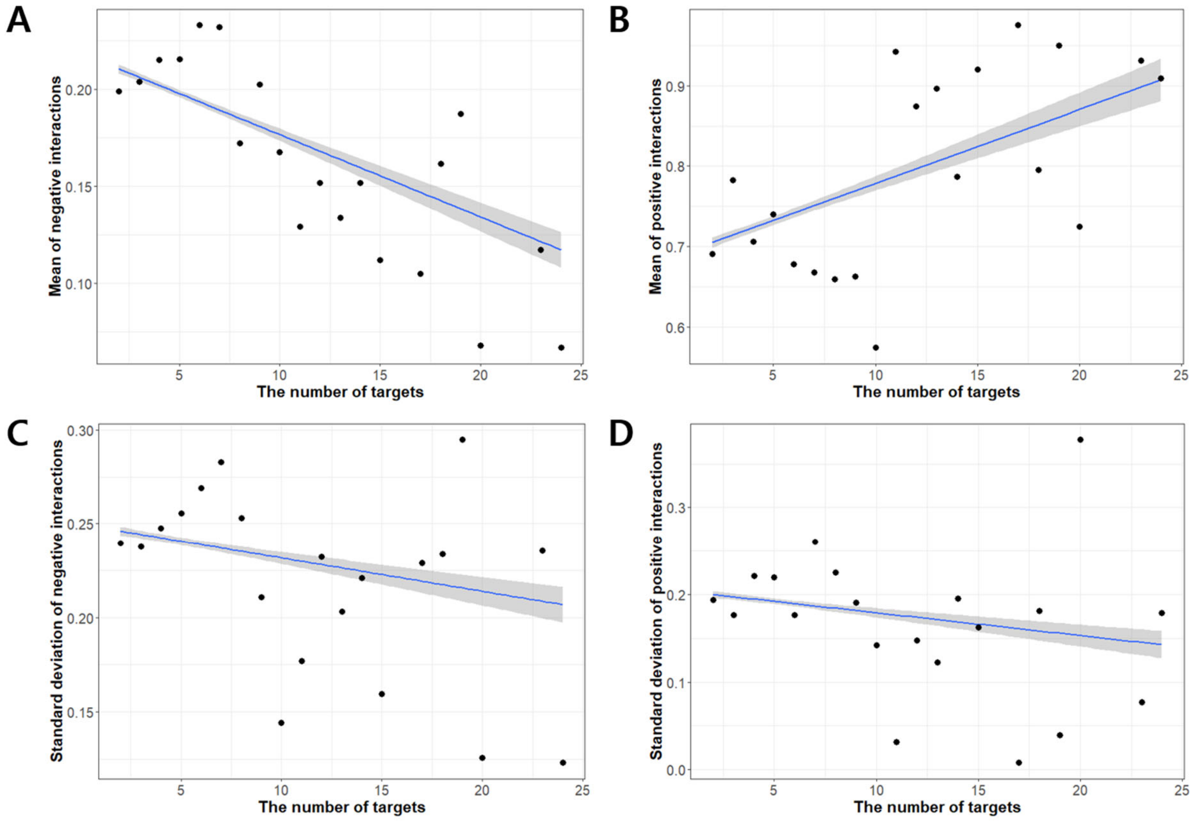

Tables

Table S1. The results of a grid search for hyperparameter tuning

(NR: negative interaction ratio, LR: learning rate)

NR	# of residual blocks	# of hidden layers	LR	AUC	Precision	Recall	R1
1	r1	c1	1.00E-05	0.932471	0.826392	0.894626	0.85813
1	r1	c2	1.00E-05	0.927289	0.827619	0.889933	0.856906
1	r1	c3	1.00E-05	0.927201	0.811585	0.918656	0.86051
1	r2	c1	1.00E-05	0.923857	0.825187	0.884935	0.851628
1	r3	c1	1.00E-05	0.921604	0.817668	0.904994	0.857801
1	r1	c3	0.001	0.920904	0.802823	0.905444	0.850055
1	r1	c2	0.0001	0.920206	0.812125	0.910558	0.857762
1	r1	c1	0.0001	0.918532	0.824653	0.881822	0.850953
1	r1	c2	0.001	0.917609	0.808709	0.89152	0.847297
1	r1	c2	0.01	0.917021	0.787374	0.905077	0.840552
1	r1	c3	0.0001	0.916465	0.825663	0.87697	0.848642
1	r1	c1	0.02	0.91233	0.775371	0.913973	0.838449
1	r1	c1	0.001	0.909227	0.793371	0.884413	0.835679
1	r3	c1	0.001	0.908356	0.812952	0.887367	0.847862
1	r3	c1	0.0001	0.908345	0.818794	0.879187	0.847241
1	r1	c1	0.03	0.903197	0.789644	0.889282	0.83513
1	r2	c1	0.0001	0.897769	0.808715	0.859275	0.831366
1	r1	c1	0.01	0.891209	0.79412	0.869358	0.828999
1	r1	c1	0.05	0.867418	0.771609	0.861743	0.8098
1	r2	c1	0.02	0.865146	0.766643	0.849947	0.802431
1	r2	c1	0.03	0.828118	0.744509	0.817262	0.777248
1	r2	c1	0.01	0.82033	0.728463	0.879826	0.787677
1	r2	c1	0.05	0.81974	0.766667	0.795521	0.774553
1	r1	c3	0.01	0.808585	0.746977	0.878252	0.786362
1	r2	c1	0.1	0.791997	0.747647	0.747447	0.738396
1	r2	c1	0.001	0.786242	0.663499	0.852537	0.737539
1	r1	c2	0.02	0.741596	0.649713	0.924744	0.739342
1	r1	c3	0.03	0.681253	0.595427	0.920917	0.698858
1	r1	c2	0.03	0.655521	0.573864	0.955106	0.697535
1	r3	c1	0.01	0.650276	0.568168	0.851759	0.668124
1	r1	c3	0.02	0.640997	0.586611	0.924152	0.700117
1	r1	c1	0.1	0.640514	0.56378	0.885068	0.677473
1	r3	c1	0.1	0.570137	0.510198	0.934465	0.652157

1	r3	c1	0.05	0.565683	0.505067	0.905587	0.642141
1	r3	c1	0.02	0.551761	0.496095	0.929973	0.642041
1	r3	c1	0.03	0.531102	0.499655	0.931553	0.643156
1	r1	c2	0.05	0.503074	0.436471	0.99881	0.607113
1	r1	c3	0.05	0.5	0.434885	1	0.605822
1	r1	c2	0.1	0.5	0.434885	1	0.605822
1	r1	c3	0.1	0.5	0.434885	1	0.605822
3	r1	c1	1.00E-05	0.943706	0.81209	0.839101	0.823858
3	r1	c3	1.00E-05	0.942487	0.803083	0.825827	0.81254
3	r1	c2	0.0001	0.941875	0.804924	0.816389	0.804004
3	r1	c2	1.00E-05	0.940564	0.821831	0.812932	0.815158
3	r1	c2	0.001	0.940121	0.783153	0.817169	0.799002
3	r1	c3	0.0001	0.940068	0.786411	0.826059	0.799275
3	r1	c1	0.0001	0.938988	0.785995	0.829877	0.805436
3	r3	c1	1.00E-05	0.937849	0.798651	0.827959	0.810927
3	r1	c3	0.001	0.937791	0.787471	0.815241	0.797292
3	r1	c1	0.001	0.937194	0.772942	0.830989	0.797765
3	r1	c2	0.01	0.935108	0.766503	0.82226	0.791067
3	r1	c3	0.01	0.935074	0.783346	0.807174	0.791435
3	r1	c1	0.01	0.932419	0.766716	0.807021	0.783898
3	r2	c1	1.00E-05	0.931794	0.794234	0.824107	0.807569
3	r3	c1	0.0001	0.928003	0.771726	0.826885	0.794407
3	r2	c1	0.0001	0.905345	0.789107	0.7542	0.769461
3	r1	c1	0.02	0.904068	0.742878	0.79006	0.757084
3	r2	c1	0.02	0.864253	0.672114	0.766208	0.703881
3	r1	c1	0.03	0.863084	0.681932	0.809686	0.722259
3	r3	c1	0.001	0.862274	0.677573	0.790801	0.721117
3	r2	c1	0.01	0.85908	0.660505	0.745831	0.691798
3	r2	c1	0.03	0.858404	0.679491	0.739261	0.691944
3	r1	c2	0.02	0.830481	0.640457	0.839148	0.689346
3	r2	c1	0.001	0.816692	0.541422	0.834423	0.640701
3	r1	c1	0.05	0.79724	0.587166	0.84548	0.661375
3	r2	c1	0.05	0.791975	0.560063	0.785435	0.63338
3	r2	c1	0.1	0.778021	0.507507	0.755283	0.596872
3	r1	c1	0.1	0.683696	0.477211	0.84662	0.552073
3	r1	c3	0.02	0.636371	0.409409	0.90461	0.510565
3	r1	c2	0.03	0.627519	0.397885	0.937669	0.500324
3	r3	c1	0.03	0.621746	0.351262	0.831091	0.477858
3	r3	c1	0.05	0.596756	0.328275	0.829356	0.461001
3	r3	c1	0.01	0.588414	0.375362	0.78438	0.479579

3	r3	c1	0.02	0.575067	0.348887	0.858793	0.471546
3	r1	c3	0.03	0.5	0.239905	1	0.386602
3	r1	c2	0.05	0.5	0.239905	1	0.386602
3	r1	c3	0.05	0.5	0.239905	1	0.386602
3	r1	c2	0.1	0.5	0.239905	1	0.386602
3	r1	c3	0.1	0.5	0.239905	1	0.386602
3	r3	c1	0.1	0.477306	0.250192	0.971951	0.396438
5	r1	c1	1.00E-05	0.944054	0.809887	0.772708	0.788968
5	r1	c3	1.00E-05	0.939839	0.78604	0.783761	0.779666
5	r1	c2	1.00E-05	0.938895	0.802576	0.756952	0.775756
5	r1	c3	0.0001	0.937828	0.765155	0.737143	0.749512
5	r1	c2	0.0001	0.937644	0.756559	0.750934	0.749332
5	r2	c1	1.00E-05	0.934491	0.807105	0.764659	0.783054
5	r1	c1	0.0001	0.933408	0.771598	0.740739	0.754311
5	r1	c2	0.001	0.932863	0.728586	0.736415	0.729915
5	r1	c3	0.001	0.929507	0.730164	0.726313	0.726542
5	r1	c1	0.001	0.929289	0.721523	0.753408	0.733475
5	r3	c1	1.00E-05	0.928072	0.77596	0.761685	0.766463
5	r1	c2	0.01	0.925388	0.728101	0.722417	0.723867
5	r1	c3	0.01	0.925123	0.671544	0.757502	0.710094
5	r1	c1	0.01	0.923855	0.760369	0.705261	0.730049
5	r1	c1	0.02	0.91524	0.726816	0.718112	0.719418
5	r3	c1	0.0001	0.912607	0.753931	0.705561	0.72403
5	r2	c1	0.0001	0.912261	0.777655	0.744188	0.757434
5	r1	c2	0.02	0.874597	0.656602	0.747122	0.668664
5	r2	c1	0.02	0.85789	0.519005	0.736267	0.588126
5	r2	c1	0.01	0.849237	0.494558	0.759946	0.582076
5	r1	c1	0.03	0.84903	0.636595	0.718642	0.644379
5	r3	c1	0.001	0.847025	0.666796	0.611745	0.622395
5	r2	c1	0.03	0.843375	0.558275	0.681234	0.598428
5	r2	c1	0.001	0.842388	0.505835	0.700979	0.571595
5	r2	c1	0.05	0.819822	0.544396	0.685439	0.587948
5	r1	c1	0.1	0.750132	0.514076	0.721281	0.536089
5	r2	c1	0.1	0.749576	0.398327	0.705923	0.483289
5	r1	c3	0.02	0.706566	0.444844	0.862147	0.496173
5	r3	c1	0.01	0.684709	0.37769	0.720996	0.447734
5	r1	c1	0.05	0.681363	0.366149	0.818306	0.453152
5	r1	c2	0.03	0.63862	0.334522	0.885135	0.413143
5	r3	c1	0.02	0.587489	0.261884	0.780918	0.352904
5	r3	c1	0.03	0.574468	0.226261	0.833642	0.347747

5	r3	c1	0.05	0.571982	0.229382	0.828579	0.350117
5	r3	c1	0.1	0.522885	0.205634	0.77926	0.307877
5	r1	c3	0.03	0.508103	0.164901	0.987654	0.281828
5	r1	c2	0.05	0.5	0.159961	1	0.275577
5	r1	c3	0.1	0.5	0.159961	1	0.275577
5	r1	c2	0.1	0.498104	0.159961	1	0.275577
5	r1	c3	0.05	0.484356	0.160155	0.995833	0.27574

Table S2. Performance evaluation of support vector machine for when heterogeneous information was available for unseen drugs.

Ratio of interactions		1:1			
Method		AUC	Precision	Recall	F1
Baseline		0.799111	0.682857	0.798829	0.735314
Single info	+DDI	0.799099	0.683158	0.797814	0.735087
	+DSE	0.799163	0.683301	0.797808	0.735114
	+DDIS	0.800202	0.704144	0.776055	0.736305
	+PPI	0.799169	0.682754	0.799337	0.735444
	+PSIM	0.799219	0.683201	0.798829	0.735508
	+PDIS	0.824909	0.746062	0.76901	0.755372
Multiple info	SVM	0.879586	0.788946	0.81761	0.801233
Ratio of interactions		1:3			
Method		AUC	Precision	Recall	F1
Baseline		0.820509	0.645462	0.653387	0.646184
Single info	+DDI	0.820744	0.64601	0.652852	0.646217
	+DSE	0.820309	0.652871	0.646281	0.646263
	+DDIS	0.821909	0.658489	0.647371	0.649057
	+PPI	0.820768	0.648131	0.64975	0.646181
	+PSIM	0.820713	0.647365	0.652838	0.646831
	+PDIS	0.83907	0.682382	0.661602	0.666139
Multiple info	SVM	0.886218	0.720545	0.736497	0.725921
Ratio of interactions		1:5			
Method		AUC	Precision	Recall	F1
Baseline		0.825509	0.648624	0.581015	0.611316
Single info	+DDI	0.825923	0.638151	0.589126	0.610346
	+DSE	0.825068	0.646137	0.583624	0.611543
	+DDIS	0.827283	0.650631	0.580555	0.610166
	+PPI	0.826438	0.648967	0.58548	0.613456
	+PSIM	0.826253	0.661493	0.578647	0.614745

	+PDIS	0.849284	0.663168	0.631706	0.642645
Multiple info	SVM	0.879606	0.691871	0.665918	0.674268

Table S3. Performance evaluation of random forest classifier for when heterogeneous information was available for unseen drugs.

Ratio of interactions		1:1			
Method		AUC	Precision	Recall	F1
Baseline		0.908904	0.864132	0.809433	0.834532
Single info	+DDI	0.905945	0.846137	0.818886	0.829051
	+DSE	0.902251	0.852507	0.796567	0.821047
	+DDIS	0.900215	0.833305	0.807169	0.818441
	+PPI	0.909572	0.85026	0.817994	0.830842
	+PSIM	0.912648	0.838787	0.843793	0.84083
	+PDIS	0.914357	0.869051	0.824618	0.845669
Multiple info	RF	0.917781	0.855828	0.833839	0.843891
Ratio of interactions		1:3			
Method		AUC	Precision	Recall	F1
Baseline		0.916328	0.84751	0.736633	0.786493
Single info	+DDI	0.916536	0.828952	0.752947	0.787115
	+DSE	0.910522	0.846217	0.721978	0.777484
	+DDIS	0.912666	0.836423	0.726716	0.776311
	+PPI	0.921305	0.851863	0.738379	0.789044
	+PSIM	0.925089	0.835737	0.783185	0.807201
	+PDIS	0.925283	0.808704	0.791943	0.799266
Multiple info	RF	0.928543	0.843685	0.763579	0.800734
Ratio of interactions		1:5			
Method		AUC	Precision	Recall	F1
Baseline		0.918992	0.814805	0.717162	0.761956
Single info	+DDI	0.917535	0.808035	0.721499	0.761252
	+DSE	0.911107	0.809826	0.709025	0.755561
	+DDIS	0.912987	0.811855	0.69387	0.747719
	+PPI	0.921759	0.814474	0.723807	0.76401
	+PSIM	0.924906	0.811867	0.745915	0.776707
	+PDIS	0.928778	0.823568	0.727703	0.771222
Multiple info	RF	0.932531	0.832628	0.721351	0.771666

Table S4. Performance evaluation of support vector machine for when heterogeneous information was predicted for unseen drugs.

Ratio of interactions		1:1			
Method		AUC	Precision	Recall	F1
Baseline		0.799111	0.682857	0.798829	0.735314
Single info	+DDI	0.877584	0.79082	0.815307	0.800301
	+DSE	0.877179	0.790703	0.814758	0.799995
	+DDIS	0.877252	0.787132	0.817837	0.800213
	+PDIS	0.800382	0.703854	0.777035	0.736515
Multiple info	SVM (predicted all)	0.799505	0.694274	0.785589	0.735707
	SVM (available PDIS)	0.824886	0.746659	0.76891	0.755287
Ratio of interactions		1:3			
Method		AUC	Precision	Recall	F1
Baseline		0.820509	0.645462	0.653387	0.646184
Single info	+DDI	0.884844	0.72201	0.733369	0.725193
	+DSE	0.884137	0.722221	0.734399	0.725692
	+DDIS	0.884171	0.731305	0.729654	0.726161
	+PDIS	0.82203	0.651357	0.651709	0.648614
Multiple info	SVM (predicted all)	0.820618	0.651115	0.645644	0.645649
	SVM (available PDIS)	0.839214	0.68407	0.660514	0.666499
Ratio of interactions		1:5			
Method		AUC	Precision	Recall	F1
Baseline		0.825509	0.648624	0.581015	0.611316
Single info	+DDI	0.879013	0.694254	0.664191	0.67492
	+DSE	0.878029	0.694526	0.663165	0.674317
	+DDIS	0.878255	0.699888	0.65677	0.674054
	+PDIS	0.827331	0.646683	0.580717	0.609702
Multiple info	SVM (predicted all)	0.827569	0.653132	0.583617	0.614594
	SVM (available PDIS)	0.850956	0.663651	0.634768	0.644478

Table S5. Performance evaluation of random forest classifier for when heterogeneous information was predicted for unseen drugs.

Ratio of interactions		1:1			
Method		AUC	Precision	Recall	F1
Baseline		0.908904	0.864132	0.809433	0.834532
Single info	+DDI	0.914468	0.864983	0.820332	0.841099

	+DSE	0.912936	0.846176	0.840375	0.842372
	+DDIS	0.909743	0.838872	0.833223	0.834898
	+PDIS	0.912595	0.861587	0.814234	0.836012
Multiple info	RF (predicted all)	0.903042	0.83171	0.833935	0.832087
	RF (available PDIS)	0.905663	0.841737	0.827045	0.832386
Ratio of interactions		1:3			
Method		AUC	Precision	Recall	F1
Baseline		0.916328	0.84751	0.736633	0.786493
Single info	+DDI	0.92565	0.825202	0.774701	0.798719
	+DSE	0.924028	0.821553	0.766244	0.791927
	+DDIS	0.91905	0.822096	0.755657	0.785849
	+PDIS	0.918479	0.815295	0.76994	0.79017
Multiple info	RF (predicted all)	0.916227	0.812041	0.756622	0.782744
	RF (available PDIS)	0.919797	0.808303	0.764455	0.784009
Ratio of interactions		1:5			
Method		AUC	Precision	Recall	F1
Baseline		0.918992	0.814805	0.717162	0.761956
Single info	+DDI	0.925666	0.822507	0.723749	0.768791
	+DSE	0.922551	0.816448	0.708225	0.756321
	+DDIS	0.918992	0.814805	0.717162	0.761956
	+PDIS	0.922856	0.806438	0.721951	0.760396
Multiple info	RF (predicted all)	0.916541	0.788868	0.711792	0.746235
	RF (available PDIS)	0.917656	0.792036	0.710437	0.745879

Table S6. Performance of HIDTI and NeoDTI for removing redundant DTIs

1:1						
Method			AUC	Precision	Recall	F1
HIDTI	Baseline		0.777756	0.668321	0.795919	0.720834
	Available heterogeneous information		0.877683	0.769708	0.837926	0.798879
	Predicted heterogeneous information	all	0.83093	0.721118	0.817179	0.762699
		available PDIS	0.846752	0.755512	0.81505	0.781303
NeoDTI			0.783166	0.657224	0.789271	0.7269
1:3						
Method			AUC	Precision	Recall	F1
HIDTI	Baseline		0.802944	0.611988	0.673552	0.629184
	Available heterogeneous information		0.880864	0.708745	0.726974	0.712993

	Predicted heterogeneous information	all	0.818101	0.673657	0.718365	0.677896
		available PDIS	0.8318	0.69688	0.711675	0.692621
NeoDTI			0.777501	0.578147	0.62891	0.601644
1:5						
Method			AUC	Precision	Recall	F1
HIDTI	Baseline		0.810663	0.604673	0.568588	0.565886
	Available heterogeneous information		0.890338	0.668779	0.69267	0.678541
	Predicted heterogeneous information	all	0.815068	0.634909	0.635243	0.62697
		available PDIS	0.841484	0.641555	0.652218	0.640857
NeoDTI			0.796783	0.498999	0.611472	0.54744

Table S7. Performance of SVM and RF for removing redundant DTIs

1:1						
Method			AUC	Precision	Recall	F1
SVM	Baseline		0.750409	0.627457	0.80781	0.700869
	Available heterogeneous information		0.831842	0.736942	0.80125	0.762314
	Predicted heterogeneous information	all	0.750027	0.627755	0.813628	0.7028
		available PDIS	0.773972	0.668029	0.77666	0.710582
RF	Baseline		0.8584	0.792886	0.78435	0.786686
	Available heterogeneous information		0.866557	0.778345	0.809279	0.789344
	Predicted heterogeneous information	all	0.858629	0.782273	0.809722	0.793965
		available PDIS	0.856346	0.756167	0.820395	0.785314
1:3						
Method			AUC	Precision	Recall	F1
SVM	Baseline		0.763894	0.594938	0.595547	0.580751
	Available heterogeneous information		0.834924	0.658146	0.677872	0.662673
	Predicted heterogeneous information	all	0.764108	0.573303	0.598218	0.577799
		available PDIS	0.786947	0.659131	0.587831	0.610113
RF	Baseline		0.861852	0.715173	0.668075	0.68344
	Available heterogeneous information		0.88064	0.716686	0.735993	0.721028
		all	0.873678	0.728388	0.703485	0.710596

	Predicted heterogeneous information	available PDIS	0.873899	0.706188	0.716368	0.705773
1:5						
Method			AUC	Precision	Recall	F1
SVM	Baseline		0.75989	0.595026	0.49283	0.528599
	Available heterogeneous information		0.826558	0.600786	0.633443	0.610219
	Predicted heterogeneous information	all	0.761579	0.580665	0.537597	0.531937
		available PDIS	0.791432	0.64748	0.532176	0.567448
RF	Baseline		0.863659	0.706661	0.625022	0.654697
	Available heterogeneous information		0.882593	0.713362	0.645801	0.670527
	Predicted heterogeneous information	all	0.874219	0.707761	0.631296	0.655534
		available PDIS	0.868967	0.697324	0.633223	0.65426

Table S8. Performance of HIDTI based on drug categories.

Code	Contents	Average AUC	# of interactions
A	Alimentary tract and metabolism	0.915	1275
B	Blood and blood forming organs	0.635	66
C	Cardiovascular system	0.917	3403
D	Dermatologicals	0.761	66
G	Genito-urinary system and sex hormones	0.891	210
H	Systemic hormonal preparations, excluding sex hormones and insulins	1	10
J	Antiinfectives for systemic use	0.406	2701
L	Antineoplastic and immunomodulating agents	0.733	2016
M	Musculo-skeletal system	0.828	351

N	Nervous system	0.948	11325
P	Antiparasitic products, insecticides and repellents	0.661	91
R	Respiratory system	0.954	253
S	Sensory organs	0.948	105
V	Various	0.875	66

Table S9. Performance of HIDTI with available heterogeneous information after ablating heterogeneous features.

Ratio of interactions	1:1				
	AUC	BCE	Precision	Recall	F1
HIDTI-DDI	0.91767	0.43772	0.85169	0.84749	0.84926
HIDTI-DSE	0.91652	0.4259	0.83858	0.85812	0.84747
HIDTI-DDIS	0.914	0.42095	0.85372	0.84716	0.84991
HIDTI - (all drug-related)	0.90975	0.3963	0.83289	0.84884	0.8404
HIDTI-PPI	0.91032	0.4589	0.81549	0.86161	0.83645
HIDTI-PSIM	0.90736	0.56408	0.82533	0.84288	0.83167
HIDTI-PDIS	0.91095	0.45189	0.82936	0.8466	0.83736
HIDTI - (all protein-related)	0.88651	0.46609	0.78846	0.84291	0.81306
Ratio of interactions	1:3				
	AUC	BCE	Precision	Recall	F1
HIDTI-DDI	0.93247	0.34914	0.83371	0.79079	0.80957
HIDTI-DSE	0.9356	0.32471	0.81419	0.82475	0.8169
HIDTI-DDIS	0.93053	0.34572	0.83482	0.78946	0.81066
HIDTI - (all drug-related)	0.92957	0.29459	0.80288	0.81487	0.80776
HIDTI-PPI	0.93343	0.32369	0.77799	0.83175	0.80226
HIDTI-PSIM	0.9266	0.3462	0.84415	0.79922	0.81842
HIDTI-PDIS	0.92518	0.35428	0.80409	0.79402	0.79776
HIDTI - (all protein-related)	0.90429	0.34971	0.78847	0.77156	0.77609
Ratio of interactions	1:5				
	AUC	BCE	Precision	Recall	F1
HIDTI-DDI	0.93547	0.26227	0.81242	0.7453	0.77371
HIDTI-DSE	0.93525	0.25287	0.78184	0.76754	0.77373
HIDTI-DDIS	0.93226	0.24682	0.81651	0.74764	0.7787
HIDTI - (all drug-related)	0.92753	0.23021	0.78951	0.73596	0.76038
HIDTI-PPI	0.92857	0.27608	0.73649	0.75055	0.74153
HIDTI-PSIM	0.92521	0.33314	0.775	0.74122	0.75414

HIDTI-PDIS	0.9244	0.28231	0.73425	0.74878	0.739
HIDTI - (all protein-related)	0.90101	0.28061	0.74887	0.70324	0.72363

Table S10. Performance of HIDTI with predicted heterogeneous information after ablating heterogeneous features.

Ratio of interactions	1:1				
	AUC	BCE	Precision	Recall	F1
HIDTI-DDI	0.87701	0.57381	0.78856	0.82419	0.80323
HIDTI-DSE	0.88463	0.53849	0.80094	0.81773	0.80791
HIDTI-DDIS	0.8905	0.48106	0.82096	0.81945	0.81793
HIDTI - (all drug-related)	0.90761	0.40905	0.82041	0.8534	0.83404
HIDTI-PPI	0.86701	0.56235	0.76023	0.82735	0.79007
HIDTI-PSIM	0.87247	0.57557	0.7938	0.80299	0.79619
HIDTI-PDIS	0.87894	0.55124	0.79287	0.80985	0.79968
HIDTI - (all protein-related)	0.86121	0.56821	0.77308	0.79173	0.78063
Ratio of interactions	1:3				
	AUC	BCE	Precision	Recall	F1
HIDTI-DDI	0.88586	0.45151	0.81948	0.70962	0.75798
HIDTI-DSE	0.89557	0.39523	0.81285	0.73731	0.77157
HIDTI-DDIS	0.8988	0.38976	0.80964	0.75295	0.77643
HIDTI - (all drug-related)	0.91721	0.31838	0.81426	0.78866	0.80055
HIDTI-PPI	0.88225	0.41943	0.80109	0.70797	0.74981
HIDTI-PSIM	0.88596	0.42548	0.79161	0.71489	0.74842
HIDTI-PDIS	0.88698	0.44564	0.80534	0.7195	0.75637
HIDTI - (all protein-related)	0.87761	0.40327	0.79175	0.69553	0.73846
Ratio of interactions	1:5				
	AUC	BCE	Precision	Recall	F1
HIDTI-DDI	0.88522	0.39089	0.75032	0.66527	0.70098
HIDTI-DSE	0.89492	0.3368	0.75966	0.68511	0.71802
HIDTI-DDIS	0.8981	0.30016	0.77781	0.67924	0.72066
HIDTI - (all drug-related)	0.92753	0.23021	0.78951	0.73596	0.76038
HIDTI-PPI	0.87291	0.38024	0.74245	0.63833	0.6849
HIDTI-PSIM	0.87907	0.36881	0.75062	0.65651	0.69928
HIDTI-PDIS	0.88042	0.35535	0.76164	0.65182	0.70143
HIDTI - (all protein-related)	0.86214	0.35354	0.73844	0.62195	0.67131

Table S11. Top 10 predictions for unseen drugs according to protein classes

class	drug_id	protein_id	label	prediction
Enzyme	DB00580	P35354	1	0.975852
	DB00461	P35354	1	0.971565
	DB00480	P35354	1	0.970382
	DB00554	P35354	1	0.969807
	**DB04572**	**P35354**	0	0.969181
	DB00712	P35354	1	0.969097
	DB00861	P35354	1	0.968978
	DB00814	P35354	1	0.968322
	DB00963	P35354	1	0.968048
	DB00991	P35354	1	0.967143
Transporter	DB00543	P14867	1	0.985069
	DB00475	P14867	1	0.985059
	DB00794	P14867	1	0.984752
	DB00231	P14867	1	0.98387
	**DB00398**	**P43681**	0	0.981746
	DB00349	P14867	1	0.981221
	DB00842	P14867	1	0.980784
	**DB06216**	**P14867**	0	0.979905
	DB01068	P14867	1	0.97887
	DB01198	P14867	1	0.976128
G-protein coupled receptor	DB00964	P08913	1	0.985744
	DB00363	P11229	1	0.985111
	DB00697	P08913	1	0.984952
	DB00543	P11229	1	0.984598
	DB00246	P11229	1	0.984545
	DB00484	P08913	1	0.984369
	DB00622	P11229	1	0.984339
	DB00575	P08913	1	0.984168
	**DB00277**	**P08913**	0	0.983864
	DB00800	P08913	1	0.983093
Voltage-gated ion channel	**DB01119**	**Q09470**	0	0.888796
	**DB00706**	**Q9NY46**	0	0.881651
	DB01119	Q12791	1	0.816415
	DB00356	Q12791	1	0.801233
	DB01189	Q09470	1	0.781274
	DB05246	O43497	1	0.779728
	DB00999	Q12791	1	0.75915

	DB00909	O43497	1	0.757438
	DB00228	Q12791	1	0.738544
	**DB01204**	**Q9NR82**	0	0.735515
Transcription factor	**DB00819**	**P04150**	0	0.972747
	DB00351	P04150	1	0.956148
	DB00860	P04150	1	0.955847
	**DB01097**	**P03372**	0	0.952076
	DB01013	P04150	1	0.950431
	DB00896	P04150	1	0.950218
	**DB01173**	**P04150**	0	0.949509
	DB01130	P04150	1	0.948963
	DB00324	P04150	1	0.948047
	DB00635	P04150	1	0.947706
